# Shared sorrow, shared costs: cost-effectiveness analysis of the *Empowerment* group therapy approach to treat affective disorders in refugee populations

**DOI:** 10.1192/bjo.2023.504

**Published:** 2023-06-22

**Authors:** Michael Strupf, Andreas Hoell, Malek Bajbouj, Kerem Böge, Maren Wiechers, Carine Karnouk, Inge Kamp-Becker, Tobias Banaschewski, Andreas Meyer-Lindenberg, Michael Rapp, Alkomiet Hasan, Peter Falkai, Ute Habel, Andreas Heinz, Paul Plener, Franziska Kaiser, Stefanie Weigold, Nassim Mehran, Aline Übleis, Frank Padberg

**Affiliations:** Department of Psychiatry and Psychotherapy, LMU University Hospital, Munich, Germany; Department of Psychiatry and Psychotherapy, Central Institute for Mental Health, Medical Faculty Mannheim/ University of Heidelberg, Mannheim, Germany; Department of Psychiatry and Psychotherapy, Charité - Universitätsmedizin, Campus Benjamin Franklin, Berlin, Germany; Department of Child and Adolescent Psychiatry, Philipps-University Marburg, Marburg, Germany; Department of Child and Adolescent Psychiatry and Psychotherapy, Central Institute of Mental Health, Medical Faculty Mannheim, University of Heidelberg, Mannheim, Germany; Social and Preventive Medicine, University of Potsdam, Potsdam, Germany; Department of Psychiatry, Psychotherapy and Psychosomatic, Medical Faculty, University of Augsburg, Augsburg, Germany; Department of Psychiatry and Psychotherapy, RWTH Aachen University, Aachen, Germany; Department of Psychiatry and Psychotherapy, Charité - Universitätsmedizin, Campus Mitte, Berlin, Germany; Department of Child and Adolescent Psychiatry and Psychotherapy, University of Ulm, Ulm, Germany; and Department of Child and Adolescent Psychiatry, Medical University of Vienna, Vienna, Austria

**Keywords:** Cost-effectiveness, cost-utility, group therapy, refugees, affective disorders

## Abstract

**Background:**

Refugees and asylum seekers (RAS) in Germany need tailored and resource-oriented mental healthcare interventions.

**Aims:**

To evaluate the cost-effectiveness of group psychotherapy for RAS with moderate depressive symptoms.

**Method:**

This is a *post hoc* cost-effectiveness analysis of *Empowerment* group psychotherapy that was embedded in a stratified stepped and collaborative care model (SCCM) from the multicentre randomised controlled MEHIRA trial. One hundred and forty-nine participants were randomly assigned to SCCM or treatment as usual (TAU) and underwent *Empowerment* (i.e. level 3 of the SCCM for adults) or TAU. Effects were measured with the nine-item Patient Health Questionnaire (PHQ-9) and quality adjusted life-years (QALY) post-intervention. Health service and intervention costs were measured. Incremental cost-effectiveness ratios (ICER) were estimated and net monetary benefit (NMB) regressions with 95% confidence intervals were performed. Cost-effectiveness was ascertained for different values of willingness to pay (WTP) using cost-effectiveness acceptability curves for probable scenarios. Trial registration number: NCT03109028 on ClinicalTrials.gov.

**Results:**

Health service use costs were significantly lower for *Empowerment* than TAU after 1 year. Intervention costs were on average €409.6. *Empowerment* led to a significant change in PHQ-9 scores but not QALY. Bootstrapped mean ICER indicated cost-effectiveness according to PHQ-9 and varied considerably for QALY in the base case. NMB for a unit reduction in PHQ-9 score at WTP of €0 was €354.3 (€978.5 to −€269.9). Results were confirmed for different scenarios and varying WTP thresholds.

**Conclusions:**

The *Empowerment* intervention was cost-effective in refugees with moderate depressive symptoms regarding the clinical outcome and led to a reduction in direct healthcare consumption. Concerning QALYs, there was a lack of confidence that *Empowerment* differed from TAU.

According to the UN refugee agency, Germany hosted around 1.3 million refugees and asylum seekers (RAS) at the end of 2021.^1^ Recent meta-analytic data for 12 002 RAS indicated pooled prevalence rates of 29.9% for trauma-related symptoms and 39.8% for depressive symptoms.^2^ According to contemporary frameworks of refugee experience, mental health is both an outcome of various interdependent pre-, peri- and post-displacement stressors and represents a prerequisite for psychosocial well-being, social participation and successful integration within the host country.^3,4^ Although a large number of publications have acknowledged mental health needs in RAS, there is insufficient provision of accessible and culturally sensitive mental health services.^5,6^ In addition, RAS encounter multiple barriers to accessing mental health resources within the German healthcare system compared with those experienced by German residents, including long waiting times for specialised services, reimbursement of costs for interpreters and transport, limited access to psychotherapy owing to legislation and insufficient cooperation between service providers.^7–9^ Unmet mental health needs may entail the risk of symptom deterioration and chronic courses, leading to insufficient social participation, work absenteeism, unemployment and poverty in RAS.^10^ From a health-economic perspective, insufficient and inefficient allocation of healthcare resources leads to situations where migrants are overrepresented in emergency care settings and forensic or acute psychiatry while being underrepresented in out-patient (subsequent) treatments, rehabilitative services and psychotherapeutic settings.^11–14^

Against this background, the Mental Health in Refugees and Asylum Seekers (MEHIRA) study was conducted between 2018 and 2020 in seven German university hospitals.^15,16^ The trial was designed to optimise resource allocation and facilitate access to healthcare for RAS by implementing a stratified stepped and collaborative care model (SCCM) for the treatment of depressive symptoms and comparing with to treatment as usual (TAU). The main analysis of pre-defined primary and secondary outcomes was published in 2022, reporting significant effects in favour of the SCCM for effectiveness and cost-effectiveness across all levels and interventions.^16^

Here, we present a *post hoc* analysis of cost-effectiveness for the group psychotherapy intervention called *Empowerment*^17^ (i.e. level 3 of the SCCM for adults) compared with TAU. Given the scarcity of tailored culturally sensitive mental health resources in the German healthcare system, we aimed to gather intervention-specific information that would reach beyond the overarching question of whether a stepped care approach is suitable for introduction in the treatment of RAS. The main publication provided evidence for the effectiveness of care coordination with smooth transitions between a set of tailored interventions adjusted for symptom severity.^16^ It did not include an in-depth evaluation of the single and innovative components currently not included in the reimbursement scheme of the mental healthcare system in Germany. There are first indications that *Empowerment* is an effective, stand-alone treatment for refugees^18,19^ that could be delivered by multiple service providers with a range of qualifications in diverse settings, for instance, practising psychotherapists in out-patient care, social workers in refugee reception centres or mental health staff in in-patient psychiatry. Thus, an (explorative) analysis of the health economical parameters is of high relevance from both clinical and healthcare providers’ perspectives. Tailored interventions for RAS such as group psychotherapy might be cost-effective^20^ while saving resources in adjacent healthcare sectors (e.g. emergency care) and reducing costs associated with future mental healthcare. The *Empowerment* intervention incorporates these requirements by designing a treatment for affective disorders that builds on existing resources and strengthens self-help skills among participants.^17^ The aim of this study was to investigate the costs and cost-effectiveness of the *Empowerment* intervention compared with TAU. Furthermore, we evaluated the impact of this target-oriented programme on the consumption of (mental) health resources by RAS after 1 year.

## Method

### Setting, design and participants

This was a *post hoc* cost-effectiveness analysis of the level 3 intervention (i.e. *Empowerment* group psychotherapy) from the multicentre randomised controlled trial (RCT) MEHIRA.^15,16^ The trial was observer-blinded, and randomisation to SCCM or TAU was performed using a 1:1 fixed block size randomisation scheme carried out before the severity of depression had been assessed for any participant. Then, participants were assigned to one of the four levels of SCCM according to their depression severity at baseline, as measured with the nine-item Patient Health Questionnaire (PHQ-9).^21^

This study was performed in accordance with the Helsinki Declaration of 1975, as revised in 2008. The ethics committee of the Ludwig-Maximilians-University Munich (approval number 17-883) and the ethics boards of the other study sites approved all procedures involving patients. Study participants were enrolled from April 2018 to December 2019. All patients gave written informed consent to their participation in this study. The MEHIRA project was registered at ClinicalTrials.gov (registration number: NCT03109028; registration date 11 April 2017).

A detailed description of the trial,^15^ the main publication on effectiveness and cost-effectiveness of the SCCM,^16^ and intervention-specific evaluations of the effectiveness^18^ and predictors of the *Empowerment* intervention^19^ are available elsewhere.

Inclusion criteria for participants of the present cost-effectiveness analysis were: (a) legal status as refugee or asylum seeker as defined by the United Nations refugee agency (UNHCR),^1^ (b) age between 18 and 65 years, (c) native language Arabic or Farsi, or fluent in English or German, and (d) PHQ-9 score between 15 and 19 points. Exclusion criteria were: (a) diagnosed with a psychotic or degenerative disorder, (b) missing informed consent, and (c) acute risk of suicidality (score ≥4 on item 10 of the Montgomery–Åsberg Depression Rating Scale^22^). Recruitment was conducted via local referring general practitioners, social workers, refugee accommodation centres and clearing houses. Eligible participants received complete information on the course, purpose, risks and requirements of the study. Professional interpreters, if required, translated information. Data collections were done at baseline (T0), post-intervention after 12 weeks (T1), and at follow-up after 24 (T2) and 48 weeks (T3). For purposes of evaluation, we analysed data according to initially randomised treatment arms, i.e. intention-to-treat (ITT).

### Treatment arms

#### Intervention (SCCM)

*Empowerment* is a culture-sensitive group psychotherapy for displaced patients with affective disorders.^17,18^ The aim of the intervention is to empower participants with knowledge and coping strategies to deal with post-migration stressors and depressive symptoms. Key interventions are psychoeducation, behavioural activation and skills acquisition. During the intervention period of 12 weeks, 16 sessions of 90 min were applied, with two sessions per week for the first four weeks and one session per week for the remaining eight weeks. Group size was intended to be between four and ten participants. *Empowerment* was conducted by German-speaking therapists with the help of Arabic- or Farsi-speaking language mediators or by native-speaking therapists. Written content was provided in Arabic, Farsi or German as required. All therapists were mental healthcare professionals with advanced or completed postgraduate clinical training and received 1 day of training in implementing the manual. Adherence to the manual was monitored through regular supervision in person and via phone. Participants in the *Empowerment* arm were not excluded from available mental healthcare services or social and psychosocial services within the respective regions.

### TAU condition

In TAU, participants were allowed to receive all available mental healthcare services and social and psychosocial services within the respective regions. The study centres themselves did not influence the type, nature, time, frequency or intensity of treatments and services available for participants in the TAU condition, nor did they regulate or stipulate providers of the corresponding treatments or services.

### Measures

#### Cost measures

We adopted a health service provider perspective including intervention costs and direct healthcare resource use costs. Direct resource use of participants was collected by self-report at each measurement point, using an adapted version of the standardised Mannheim Module Resource Use (MRU^23^). The MRU measures units of health service resources, i.e. frequency of contacts, consumed during the previous 3 months in the following areas: general practitioners, other out-patient specialists, emergencies, mental health specialists (psychologists, psychiatrists), remedies and other out-patient therapists, counselling and health support services. The MRU was used to collect days spent in in-patient general or psychiatric care. Medication was indirectly measured by visits to pharmacies, and we equated visits with submissions of drug provision sheets. We did not limit resource use related to depression care. Finally, we combined data on resource use with specific unit costs. Unit costs were derived using a mixture of opportunity costs and *per diem* costs from nationally or regionally (i.e. for each participating federal state) available data sources. The reference year for all prices in euros (€) was 2019. If necessary, we indexed prices based on the German Federal Statistical Office consumer price index^24^ (supplementary Table 1 available at available at https://doi.org/10.1192/bjo.2023.504).

We used micro-costing to determine the costs of the *Empowerment* programme. Key persons were interviewed with the help of a structured interview that collected data on recurring or annual programme expenses due to consumables, personnel and operating costs. We used the ITT sample of *Empowerment* participants to calculate the *per capita* costs of the programme (supplementary Table 2). Finally, we combined resource use costs with *Empowerment* programme costs, which we called the base case, as it was considered to be the most likely scenario in medical care for RAS. For reasons of sensitivity and robustness, we created alternative scenarios. The on-top case, i.e. the intervention costs only, included annual programme costs for the *Empowerment* condition but no resource use costs for all participants, assuming equal resource consumption in *Empowerment* and TAU after one year. The optimal case was an optimal programme utilisation scenario where *Empowerment* had an expected capacity utilisation of 100%, i.e. 16 completed *Empowerment* groups per year, with eight participants per group, equalling 128 participants per year. We did not discount or depreciate costs owing to the short time horizon of the study.

### Outcome measures

We considered incremental cost per PHQ-9 (clinical outcome) and incremental cost per quality adjusted life year (QALY, preference-based outcome) at 12-month follow-up as economic endpoints. We assessed depression severity with the self-rated scale PHQ-9 at post-intervention. The PHQ-9 records nine symptoms on a four-point Likert scale covering the past 2 weeks. The instrument showed good psychometric properties, with an internal consistency of Cronbach's α = 0.86–0.89 and test–retest reliability of 0.84^21^ and has been validated across multiple cultural backgrounds and languages.^25,26^ For the cost-utility analysis, we calculated QALY elicited from the WHO Quality of Life questionnaire, brief version (WHOQOL-BREF), a 26-item questionnaire where each item is rated on a five-point Likert scale.^27^ The tool provides an intercultural assessment of health-related quality of life that covers general, physical, psychological, social and environmental health domains.^27^ The WHOQOL-BREF is a recommended patient-reported outcome measure in mental health^28^ and is frequently used with RAS around the globe, both in epidemiological and intervention studies.^29^ Its intercultural psychometric and discriminating properties are good,^30^ and there is an Arabic version of the tool. We transferred WHOQOL-BREF scores to WHOQOL-100 values using established algorithms proposed by the WHOQOL group.^27^ To obtain utility values with a possible range from 0 to 1, we converted WHOQOL-BREF values to QALYs using an algorithm proposed by Salize and Kilian.^23^ We preferred this method of eliciting QALYs to other direct measurements because of their supposed difficulty and time-consuming nature, and to other preference-based measures because of expected ceiling effects and missing established value sets for Arabic-speaking countries. We extended QALY to a 12-month period using available data points or the last observation carried forward (LOCF) method. We adjusted PHQ-9 values and QALY for age, gender, study site and initial value. As the time horizon of the study was only 1 year, we did not discount outcomes.

### Statistical analyses

Sample descriptions were done with *t*-test statistics and χ²-tests. Statistical analyses were performed according to the ITT principle. Missing resource use and outcome data were imputed with the LOCF method, a conservative approach strengthening the null hypothesis of equal costs and effects between *Empowerment* and TAU. Owing to the highly right-skewed cost data, we log-transformed resource use costs and applied generalised linear models (GLM) with gamma distribution and identity link function to estimate differences in healthcare costs between groups.^31^ We performed adjusted models with group as the explanatory variable and age, gender, centre and baseline costs as covariates for all scenarios, because randomisation was not done at the level of depression severity. We determined the incremental cost-effectiveness ratios (ICER) that represent the additional costs to obtain one additional QALY or to decrease the PHQ score by one point. We performed non-parametric bootstrapping with 10 000 ICER replications to acknowledge ICER variability and plotted ICER replications on cost-effectiveness planes. In addition, we performed multivariate net monetary benefit (NMB) regressions as proposed by Hoch and colleagues,^32^ with group as the explanatory variable and the covariates mentioned above. The NMB approach is a function of willingness to pay (WTP) thresholds (λ). We considered different λ to check statistical uncertainty around incremental costs and effects with cost-effectiveness acceptability curves (CEAC). To satisfy the condition of parameter uncertainty, we performed cost-effectiveness and cost-utility analyses for the base case, optimal case and on-top case. All analyses were performed using SPSS (SPSS Inc., Chicago, Illinois, USA) version 26, SAS statistical software (SAS Institute Inc, Cary, North Carolina, USA) version 9.4 and Excel 2016 for Windows.

## Results

### Participants

In total, 149 of all eligible participants were classified as moderately depressed, as defined by PHQ-9 values from 15 to 19. Prior to this, 81 subjects had been randomised to SCCM and 68 to TAU. Supplementary Table 3 shows the baseline characteristics. Participants had a mean age of 32.2 (s.d. = 9.4) years, and the majority were male (61.7%), had a temporary residence permit (86.4%), lived in refugee accommodation (51.0%), were unemployed (87.5%), perceived social relegation (lower middle class to lower class in country of origin 19.7% *v*. now 67.1%) and showed concomitant post-traumatic stress disorder symptoms (64.7%). Characteristics did not differ significantly between the intervention and control group.

### Cost analyses

We identified mean *per capita* resource use baseline costs of €2798.7 (s.d. = €3602.3) per year. Resource use costs differed significantly between study sites. *Per capita* resource use costs were statistically equivalent between TAU and *Empowerment*. Nearly half of the healthcare costs were accounted for by psychological or psychiatric support (*Empowerment* = 47.7% and TAU = 44.5%). At post-intervention, mean *per capita* resource use costs were €1780.2 (s.d. = €1952.4) per year. Cost reductions went along with a decreased utilisation of psychological or psychiatric support, leading to proportions of 15% of total resource use costs in *Empowerment* and 40% in TAU. We identified resource use costs of €1401.1 (s.d. = 1697.0) per year for *Empowerment* participants and €2231.8 (s.d. = 2145.0) per year for TAU participants. Adjusted exponential regression coefficients indicated that resource use costs for *Empowerment* were 31% lower than those for TAU (Exp(B) = 0.69, 95% CI = 0.50 to 0.95, *P* = 0.024) at post-intervention (Table 1).

**Table 1:** Comparison of *per capita* costs of *Empowerment* versus TAU 1 year after baseline (base case)

	*Per capita* costs after one year in €			
	*Empowerment* (*n* = 81) mean (s.d.)	TAU (*n* = 68) mean (s.d.)	GLM-adjusted Exp(B) (95% CI)	*P*-value
Emergency care	93.3 (423.7)	37.2 (125.1)	−	
Primary care^a^	200.6 (172.7)	230.9 (218.7)	−	
Psychiatric care^b^, psychological support	211.7 (689.8)	898.8 (1,548.4)	−	
General hospital	287.8 (1,008.3)	223.4 (778.4)	−	
Other out-patient therapies and/or providers	5.2 (32.4)	40.3 (129.4)	−	
Medication	625.2 (746.7)	834.8 (993.5)	−	
Resource use costs^c^	1401.1 (1697.0)	2231.8 (2145.0)	0.69 (0.50–0.95)	0.024
*Empowerment* programme costs (base case)	409.6	0	−	
*Empowerment* programme costs (optimal case)	347.9	0	−	
Total healthcare costs (base case)	1810.7 (1.697.0)	2231.8 (2145.0)	0.97 (0.75–1.26)	0.845
Total healthcare costs (optimal case)	1749.0 (1697.0)	2231.8 (2145.0)	0.93 (0.63–1.17)	0.599

a. Includes language mediation.

b. Includes out-patient and in-patient treatment.

c. Combining resource use costs from the sections and areas mentioned above. Base case scenario, i.e. programme cost of the intention-to-treat sample and resource use cost. Generalised linear model (GLM) adjusted for age, gender, study site and baseline resource use costs. GLM values were related to log-transformed resource use costs and total costs. Exponential regression parameters and associated confidence limits are reported.

TAU, treatment as usual.

*Empowerment* programme costs are provided in supplementary Table 2. During the rollout phase of the trial, 13 groups were completed, with 81 participants overall. Based on the ITT sample, we calculated *per capita* programme costs of €409.6. We calculated optimal programme costs – considering optimal use of personnel and operating resources – of €347.9 per patient. We added programme costs to resource use costs at post-intervention for *Empowerment* participants. As a result, base case *per capita* costs were €1810.7 (Table 1), whereas optimal case *per capita* costs were €1749.0. Exponential regression coefficients of adjusted log-transformed cost values demonstrated no significant differences in total costs between randomisation groups for the base case and optimal case after 1 year.

### Cost-effectiveness analyses

The primary outcome was analysed in all 149 participants. Mean PHQ-9 at baseline was 17.0 (s.d. = 2.4) and did not differ between randomisation groups or study regions. The mean value at post-intervention was 16.6 (s.d. = 4.6). Estimated marginal group means were 15.7 (95% CI = 14.7 to 16.6) for *Empowerment* and 17.7 (95% CI = 16.6 to 18.8) for TAU. Differences at post-intervention were significant (B = −2.04, 95% CI = −3.47 to −0.61, *P* = 0.005).

**Fig. 1:**
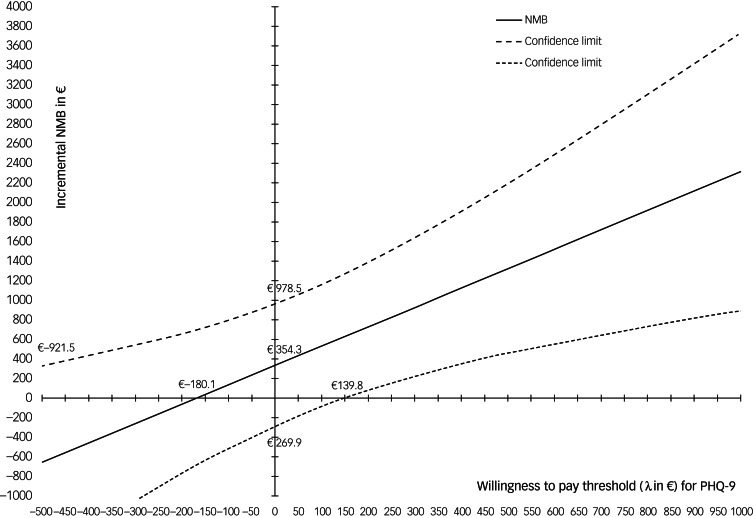
Incremental net monetary benefit (NMB) by willingness to pay for *Empowerment* versus treatment as usual on clinical outcome (Patient Health Questionnaire): estimates and statistical uncertainty for the base case scenario. Note that the incremental NMB was positive, and the x-intercept indicates the adjusted incremental cost-effectiveness ratio at an incremental value of €−180.1 with 95% CI €−921.5 to €139.8.

**Fig. 2:**
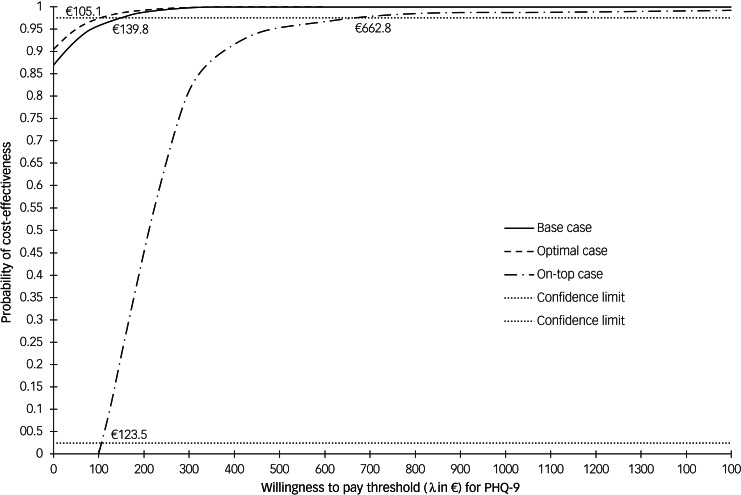
Cost-effectiveness acceptability curves based on incremental net monetary benefit (NMB) regressions for *Empowerment* versus treatment as usual (TAU) on clinical outcome (nine-item Patient Health Questionnaire; PHQ9): all three scenarios. Curves show the probability that *Empowerment* was acceptable (values on the y-axis) in relation to TAU in terms of willingness to pay for an additional one-point reduction in PHQ-9 values, given varying thresholds for willingness to pay (x-axis) based on incremental NMB regressions. The three values at the upper 97.5% confidence dotted line indicate statistical uncertainty regarding the good value of *Empowerment*.

The estimated bootstrapped ICER for the base case was €−214.6 (95%CI = €−631.2 to €87.9) and *Empowerment* clearly dominated the TAU condition with 91.2% of 10 000-bootstrap replications located in the south-east quadrant of the cost-effectiveness plane (supplementary Fig. 1) synonymous for higher effects at lower costs. We used adjusted NMB regressions to estimate the acceptability of *Empowerment* according to predefined WTP thresholds. Fig. 1 displays the NMB for a unit reduction in PHQ-9 of the base case represented by mean values (solid line) and 95%CI (dashed lines). The solid line intersects the X-axis at €-180.1 (the adjusted ICER) with a positive slope of 1.97 (the adjusted effect). Intersections of the Y-axis represent the WTP of €0 with a mean net benefit of €354.3 (solid line), but confidence limits comprise values ≤€0 (95%CI=€978.5 to €-269.9). The confidence limit to the right intersects the X-axis at €139.8, i.e. the maximum sum that has to be invested to be confident that *Empowerment* is cost-effective compared to TAU. Fig. 2 displays similar CEAC for all three scenarios. CEAC for the base case revealed that *Empowerment* obtained an additional effect without any additional costs with a probability of 0.87. CEAC for the base case, optimal case and on-top case exceeded the 97.5%CI at €139.8, at €105.1 and at €662.8 respectively, indicating cost-effectiveness at these specific maximum WTP values (Fig. 2). Likewise, the CEAC for on-top case indicated that a minimum of €123.5 had to be invested to produce an additional effect.

### Cost-utility analyses

Cost-utility analyses were performed on 136 participants (*Empowerment* = 77 and TAU = 59) who provided QALY baseline values. Missing follow-up values were imputed. Mean baseline QALY values were 0.45 (s.d. = 0.12) and did not differ between randomisation groups. At one year, there was a point difference of. 04 QALY in favour of *Empowerment*, but this difference was not statistically significant. (B = 0.04, 95%CI = −0.09 to 0.17, *P* = 0.532).

**Fig. 3:**
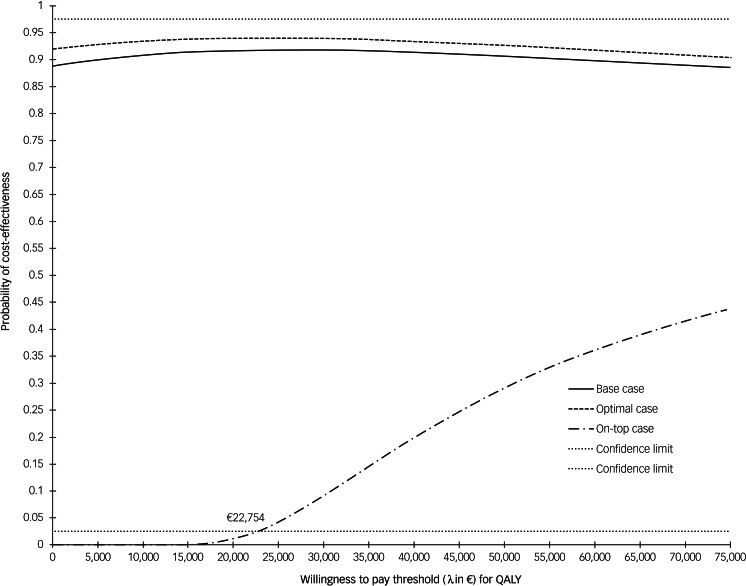
Cost-effectiveness acceptability curves based on incremental net monetary benefit (NMB) regressions for *Empowerment* versus treatment as usual (TAU) on quality-adjusted life years (QALY): all three scenarios. Curves show the probability that *Empowerment* was acceptable (values on the y-axis) in relation to TAU in terms of willingness to pay for an additional QALY, considering different thresholds for willingness to pay (x-axis) based on incremental NMB regressions. No value reached the upper 97.5% confidence dotted line, indicating no confidence regarding the good value of *Empowerment*. The on-top scenario was rejected because of values lower than €22 754.

Because of small effects and negative cost values in favour of *Empowerment* the mean bootstrapped ICER was negative with wide confidence limits (€-17 282.8, 95%CI = €-84 617.0 to €77 781.6). ICER replications were scattered across the whole cost-effectiveness plane with 57.3% of ICER replications located in the south-east quadrant indicating that *Empowerment* dominated TAU, and 35.9% of the replications located in the south-west quadrant indicating a cost-offset but lower effects (supplementary Fig. 2). We constructed incremental NMB graphs for the different scenarios with different λ, which all had in common that the upper or the lower, or both confidence limits did not intersect the X-axis. Thus, cost-effectiveness of *Empowerment* concerning QALY could not be ensured. Fig. 3 depicts CEAC for all scenarios. CEAC for the base case and optimal case showed that the probability to obtain an additional QALY without any additional costs is large with probabilities of 0.89 and 0.92, respectively. None of the three CEAC exceeded 97.5%CI for any value of λ, signifying no maximum WTP values for cost-effectiveness of *Empowerment*. While the CEAC for the optimal case and base case had a peak at around €27 000 and afterwards decreased asymptotically, the CEAC for the on-top case started to increase following a reverse exponential function. According to the CEAC for the on-top case, a minimum of €22 750 had to be invested for an additional QALY.

## Discussion

This *post hoc* study based on the MEHIRA project aimed to evaluate the cost-effectiveness of the culture-sensitive *Empowerment* intervention compared with TAU for RAS with moderate depressive symptoms. We measured incremental changes in costs and outcomes, i.e. a clinical parameter (PHQ-9) and a well-being measure deriving QALYs, against routine mental care for RAS in Germany. In addition, we evaluated the comparative net benefit (NMB). This type of comparative effectiveness research should help all stakeholders, including patients, to make informed decisions about reasonable resource allocation.^33^

We found that resources consumed in mental healthcare and associated resource use costs were significantly lower in patients receiving the *Empowerment* intervention compared with the TAU group after 1 year. Adding costs of intervention and language mediation only slightly increased the total costs of *Empowerment* and led to comparable total costs between both groups. This may suggest the efficiency of *Empowerment*. It seems that this culture-sensitive specialised mental health service had a steering effect on patients’ resource consumption. The targeted allocation of otherwise arbitrarily consumed mental health resources made these scarce resources available to other patients in need. This was because TAU was not a ‘do nothing’ scenario but represented the diverse routine mental healthcare provided for RAS, which is currently associated with insufficient and inefficient allocation of healthcare resources.^12,13^ It is conceivable that language mediators, as employed in *Empowerment*, were jointly responsible for this steering effect on resource consumption. There is evidence that communication barriers increase the inadequate use of healthcare resources.^34,35^ Our results indicate that it might be economical for health insurance schemes to bear the costs of language mediation in the medical care of RAS because of possible major savings in the reduction of inappropriate treatments. In the German healthcare system, language mediation is not usually reimbursed (for either clinical routine care or psychotherapy). In addition, our study shows the feasibility of language mediation within group psychotherapy for refugees, which leads to modified speed and content within the psychotherapy group process owing to repetition of content in two languages. Thus, future dismantling studies should address the important question of the differential effects of language and culture mediation as single components within group interventions.

Total resource use costs after 1 year of roughly €2002, recorded bottom-up on a quarterly basis, were comparable with annual top-down (billing) data for asylum-seekers from a statutory health insurance scheme in Germany (roughly €1900^36^) and from a social service office of a medium-sized city in Germany (roughly €1530^14^ ). They were also equal to the annual resource use costs for Syrian refugees of €1920 reported by a RCT using a bottom-up approach.^37^ The main cost driver in all studies was in-patient healthcare, owing to inadequate provision of or access to primary and preventive care.^14^

In addition, *Empowerment* proved to be effective in reducing depressive symptoms in RAS.^18,19^ Cost-effectiveness analysis supported the hypothesis that *Empowerment* is cost-effective compared with TAU concerning the improvement of symptoms. There was an 87% probability of obtaining an additional effect without additional costs. With a WTP €140 for an additional improvement in depressive symptoms, one could be 97.5% confident that *Empowerment* was to be preferred over TAU from the healthcare provider perspective. On the contrary, cost-utility analysis did not support the hypothesis that *Empowerment* was cost-effective. There was no substantial difference between *Empowerment* and TAU concerning generated amounts of QALYs, and cost-saving alone did not justify a finding of cost-effectiveness.

To our knowledge, only a few cost-effectiveness studies of interventions for improving mental health in RAS have been published to date.^16,39,40^ Admittedly, there is no reason to believe that evidence-based treatments developed to treat mental disorders in average populations of host countries could not also be applied to RAS. However, adjustments to existing treatment interventions are necessary, as the interventions should both adequately treat RAS with mental disorders and facilitate their access to and continuity of mental healthcare.^5,38^ Recently conducted cost-effectiveness trials in refugees showed inconsistent results.^16,39,40^ On the one hand, the primary analysis of the MEHIRA trial showed superior indicators of cost-effectiveness, concerning PHQ-9 and QALYs, for the intervention over the TAU condition.^16^ Similarly, the Problem Management Plus intervention – a brief trans-diagnostic peer-provided psychosocial intervention to reduce psychological distress in Syrian refugees in The Netherlands – seemed likely to be cost-effective in achieving significant improvements in HSCL-25 (defined as recovery) compared with TAU.^39^ On the other hand, a German RCT in Syrian refugees called SANADAK, which used a smartphone-based low-threshold self-help app to reduce post-traumatic symptoms, did not show cost-effectiveness for the intervention as a standalone application for improving QALY compared with a control group receiving psychoeducation.^40^ However, this low-cost intervention significantly reduced self-stigma without raising resource use costs.^40^

It seems challenging to elicit QALY using current generic preference measures or quality of life measures in mental healthcare and in RAS.^41^ Small effects could be explained by inappropriate preference weights, value sets, or conversion algorithms with possible ground effects in RAS, or by a failure to achieve improvements in quality of life and consumer satisfaction with interventions. We used the WHO-QoL-BREF to capture a broad impact on daily life of RAS in a host country, although the trade-off between this comprehensive measure and QALY was information loss with questionable cross-comparability.^42^ Furthermore, it seems plausible that housing or working conditions, mobility and cooperative relationships remained unaffected by *Empowerment* because of a direct governing effect owing to the health and refugee policy in Germany.

Our study had several strengths. We analysed cost-effectiveness and cost-utility in a sample of RAS that came from a nationwide multicentre RCT. High quality standards were implemented, and missing data were imputed, yielding a high-quality and robust data-set. Furthermore, we ensured the sensitivity and robustness of analyses by calculating different scenarios. However, there were also some limitations. First, we reported secondary analyses of data from a multicentre RCT that compared an SCCM approach with TAU.^16^ These *post hoc* analyses were not originally reported in the trial protocol^15^ but were pre-planned in order to investigate the effectiveness of innovative interventions at distinct steps of the SCCM. *Post hoc* data analyses do not conform to the randomisation model of statistical inference (e.g. power calculation, randomisation process). Second, the naturalistic outcome (PHQ-9) was a condition-specific outcome with limited meaning for patients. Currently, there are no benchmarks or other values known to assume that the change in depressive symptomatology comes at reasonable costs. At the current stage of the study, we found the provision of costs of a point-change in depressive symptoms to be acceptable, but we were aware of the need to change the outcome towards depression-free days based on meaningful change of PHQ-9; this will be analysed with decision-analytic models in a forthcoming publication. Third, we calculated QALY using the profile-based quality of life measure WHO-QoL-BREF and transformed its scores to elicit QALY in the form of a visual analogue scale. We wanted to comprehensively capture several important dimensions of QOL in RAS irrespective of the utility theory representing discrete preferences under conditions of uncertainty.^43^ To date, there has been only one study mapping WHO-QoL-BREF scores to the EQ-5D-5L utility index using the Japanese value set.^44^ However, WHO-QoL-BREF domain scores were largely unrelated to utility. Thus, we advise caution in interpreting the QALY results and comparing them with those of other studies. Fourth, we used a healthcare provider perspective to analyse costs and therefore only included direct healthcare costs. Cost calculations in mental health comprise additional categories such as indirect costs (i.e. cost in other sectors surrounding healthcare owing to functional, social and work-related issues) and direct non-health costs. Thus, health costs may have been underestimated. Furthermore, although it was useful to use a bottom-up approach to capture resource use, RAS may have answered the questions inconsistently owing to the unfamiliar healthcare system. Inconsistent use of the resource use measure could be limited by ensuring that RAS were interviewed by trained staff.

## Conclusion

In summary, the *Empowerment* intervention has the potential to reduce the mental health burden among RAS by simultaneously improving resource allocation and reach of service. Whereas prior studies of peer-provided interventions for refugees showed no significant reductions in healthcare utilisation or costs,^39^ *Empowerment* was cost-effective in comparison with routine care practices within the German mental healthcare system. To support decision makers in healthcare and health policy, future studies in RAS should provide meaningful patient-reported outcomes, model longer periods, and include measurements of indirect costs and direct non-health costs.

## Data Availability

The data that support the findings of this study are available from the corresponding author, A. Hoell, upon reasonable request.

## Supplementary material

For supplementary material accompanying this paper visit http://doi.org/10.1192/bjo.2023.504. click here to view supplementary material
