# Supplementary material for: Shared sorrow, shared costs: cost-effectiveness analysis of the Empowerment group therapy approach to treat affective disorders in refugee populations
Source: BJPsych Open. 2023 Jun 22;9(4):e113. doi: 10.1192/bjo.2023.504 (PMC10305100; doi:10.1192/bjo.2023.504)
Supplement: Supplementary file 1 [file S2056472423005045sup001.zip › S2056472423005045sup001.docx]

**S1.** Unit costs of different health services and data sources

| **Health Service Type** | **Unit Cost in €** | **Source/ Calculation** |
| --- | --- | --- |
| **Hospital (Inpatient)** |  | Basic Values on Hospitals in Germany 2020 (Federal Statistical Office) & Proof of Costs of Hospitals in Germany 2020 (Federal Statistical Office); calculated for every Federal state by dividing the mean adjusted costs of treatment case by the mean length of stay per case |
| **Berlin** | 686.6 |  |
| **Bavaria** | 660.1 |  |
| **North Rhine Westphalia** | 638.2 |  |
| **Hospital (Inpatient Psychiatry)** |  | Daily pro rata billings according to individual hospital PEPP pay scales as found on individual web pages of hospitals; Basis: Depression and calculated by multiplying the basic remuneration value with the pricing ratio and adding individual surcharges |
| **Berlin** | 290*(pricing ratio*days) + 127.2 |  |
| **Bavaria** | 311.1*(pricing ratio*days) + 87.5 |  |
| **North Rhine Westphalia** | 290*(pricing ratio*days) + 108.9 |  |
| **General practitioner** |  | Case value according to the quarterly remuneration report of the National Association of Statutory Health Insurance Physicians in Germany; the case value is applied to a quarterly period and is therefore applied only once |
| **Berlin** | 56.4 |  |
| **Bavaria** | 69.5 |  |
| **North Rhine Westphalia** | 65.7 |  |
| **Emergency (outpatient emergency patients)** | 141.8 | Expert report on outpatient emergencies in hospitals 2015 (Management Consult Kesterman GmbH on behalf of the German Society of Interdisciplinary Emergency Medicine); values indexed to 2019 prices |
| **Psychotherapy** | 98.2 | Single Therapy Session according to EBM-Catalogue for 2019 |
| **Remedy/ Therapies** |  | Mean value of all remedy therapies (like physical therapy, occupational therapy, speech therapy) according to the Federal Report Remedy Quick Information 2019; contact value for each Federal state |
| **Berlin** | 25.0 |  |
| **Bavaria** | 26.3 |  |
| **North Rhine Westphalia** | 29.6 |  |
| **Socio-psychiatric service** | 134.8 | Grupp 2017; national mean value on socio-psychiatric services in 2014, values indexed to 2019 prices |
| **Social counselling** | 129.7 | Grupp 2017; national mean value on counselling centers in 2014, values indexed to 2019 prices |
| **Interpreter/ Language mediation** | 55.1 | Mean contact value (fees including travel flat rate and taxes) |
| **Medication** |  | Because of difficulty to achieve information on medication use we ask for visits to pharmacies and equate visits with submission of drug provision sheets; Federal report of the Statutory Health Insurance on Drugs Quick Information 2019; submission value for each Federal state |
| **Berlin** | 130.2 |  |
| **Bavaria** | 102.9 |  |
| **North Rhine Westphalia** | 98.8 |  |
