# Supplementary material for: Shared sorrow, shared costs: cost-effectiveness analysis of the Empowerment group therapy approach to treat affective disorders in refugee populations
Source: BJPsych Open. 2023 Jun 22;9(4):e113. doi: 10.1192/bjo.2023.504 (PMC10305100; doi:10.1192/bjo.2023.504)
Supplement: Supplementary file 1 [file S2056472423005045sup001.zip › S2056472423005045sup002.docx]

**S2** Program Costs of Empowerment

| Programme Type  (Consumables, Personnel) | Unit Costs/  Amount of  Units per … | Empowerment  Base Case (n=81)  Per capita costs in € | Empowerment  Optimal Case  (n=128)  Per capita costs in € |  | |  |
| --- | --- | --- | --- | --- | --- | --- |
| Application of Empowerment Groups | | | | | | |
| Therapists/  Psychologists | €27.2/Hour  42 Units per Group |  |  | |  | |
| Interpreters | €55.1/Unit  21 Units per Group |  |  | |  | |
| Consumables  (Handouts, Flipchart, etc.) | €10.4/Group |  |  | |  | |
| Pro rata Capital Costs  (Rent, Ancillary costs) | €1.0/ Group |  |  | |  | |
| Supervision | | | | | | |
| Psychologist | €27.2/Hour  4 Units per Group |  |  | |  | |
| Psychiatrist | €50.3/Hour  2 Units per Group |  |  | |  | |
| Costs per Group | - | €2,518.2 | | |  | |
| Retraining/ Refresher | | | | | | |
| Psychologist | €27.2/Hour  8 Units per Year |  |  | |  | |
| Total per Capita Costs | - | €409.6 | €347.9 | |  | |

*Note.* Base case referred to program-based calculation of per capita costs as observed in the trial: There were 13 Empowerment groups during the trial with 81 participants. Optimal Scenario suggested an optimal number of groups and occupancy rate per year. Due to personnel and operating resources there could have been 16 Empowerment groups each with eight participants resulting in 128 participants.
