# Supplementary material for: Shared sorrow, shared costs: cost-effectiveness analysis of the Empowerment group therapy approach to treat affective disorders in refugee populations
Source: BJPsych Open. 2023 Jun 22;9(4):e113. doi: 10.1192/bjo.2023.504 (PMC10305100; doi:10.1192/bjo.2023.504)
Supplement: Supplementary file 1 [file S2056472423005045sup001.zip › S2056472423005045sup003.docx]

**S3.** Baseline characteristics of participants according to randomisation group

|  | **Mean ± SD; N/Total N (%)** | |  |
| --- | --- | --- | --- |
|  | **Empowerment** | **TAU** |  |
|  | **(n=81)** | **(n=68)** | ***P* Value** |
| Age (years) | 32.62 ± 9.08 | 31.64 ± 9.84 | .532 |
| Female gender | 35/81 (43.2) | 22/68 (32.4) | .182 |
| Education (years) | 8.80 ± 4.41 | 8.78 ± 4.71 | .975 |
| Marital status  … Single  … Married  … Divorced  …Widowed | 31/81 (38.3)  38/81 (46.9)  9/81 (11.1)  3/81 (3.7) | 30/67 (44.8)  23/67 (34.3)  10/67 (14.9)  4/67 (6.0) | .458 |
| Current refugee status  …Permanent residence permit  … Temporary residence permit  … Permanent residence permit in EU  … No legal residence permit  … Other | 3/81 (3.7)  73/81 (90.2)  1/81 (1.2)  3/81 (3.7)  1/81 (1.2) | 3/66 (4.5)  54/66 (81.8)  4/66 (6.1)  3/66 (4.5)  2/66 (3.1) | .481 |
| SES in country of origin  … Upper class  … Upper middle class  … Middle class  … Lower middle class  … Lower class | 6/78 (7.7)  15/78 (19.2)  42/78 (53.9)  12/78 (15.4)  3/78 (3.8) | 7/64 (10.9)  10/64 (15.6)  34/64 (53.2)  13/64 (20.3)  0/646 (0.0) | .461 |
| SES – current  … Upper class  … Upper middle class  … Middle class  … Lower middle class  … Lower class | 0/77 (0.0)  4/77 (5.1)  23/77 (29.9)  16/77 (20.8)  34/77 (44.2) | 2/63 (3.2)  2/63 (3.2)  15/63 (23.8)  22/63 (34.9)  22/63 (34.9) | .163 |
| Living situation  … Private flat  … Refugee accommodation  … Shared flat  .. Other | 32/81 (39.5)  40/81 (49.4)  8/81 (9.9)  1/81 (1.2) | 19/66 (28.8)  35/66 (53.0)  10/66 (15.2)  2/66 (3.0) | .370 |
| Current employment status  … Unemployed  … Protected employment  … Employee | 70/78 (89.7)  1/78 (1.3)  7/78 (9.0) | 56/66 (84.9)  2/66 (3.0)  8/66 (12.1) | .618 |
| Current use of antidepressants or antipsychotics | 31/81 (38.8) | 28/67 (41.8) | .738 |
| Current use of psychotherapy | 15/79 (19.0) | 12/66 (18.2) | .999 |
| Current PTSD symptomatology | 48/79 (60.8) | 42/60 (70.0) | .286 |

*Abbreviations.* EU = European Union, PTSD = posttraumatic stress disorder, SD = standard deviation, SES = socioeconomic status TAU = treatment as usual.
