# Supplementary figures and images for: Shared sorrow, shared costs: cost-effectiveness analysis of the Empowerment group therapy approach to treat affective disorders in refugee populations
Source: BJPsych Open. 2023 Jun 22;9(4):e113. doi: 10.1192/bjo.2023.504 (PMC10305100; doi:10.1192/bjo.2023.504)

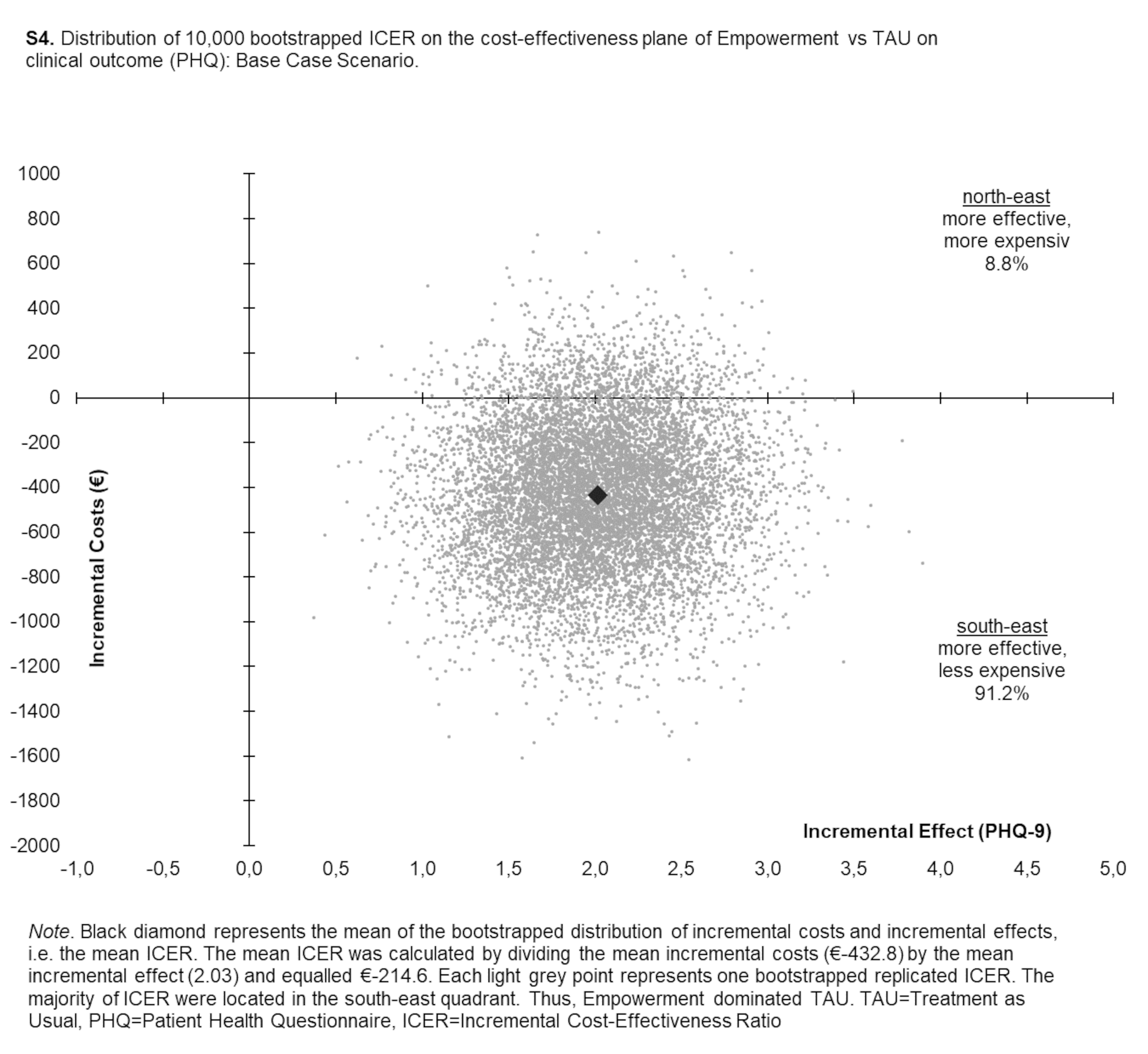

Supplement: Supplementary file 1 [file S2056472423005045sup001.zip › S2056472423005045sup004.tif]

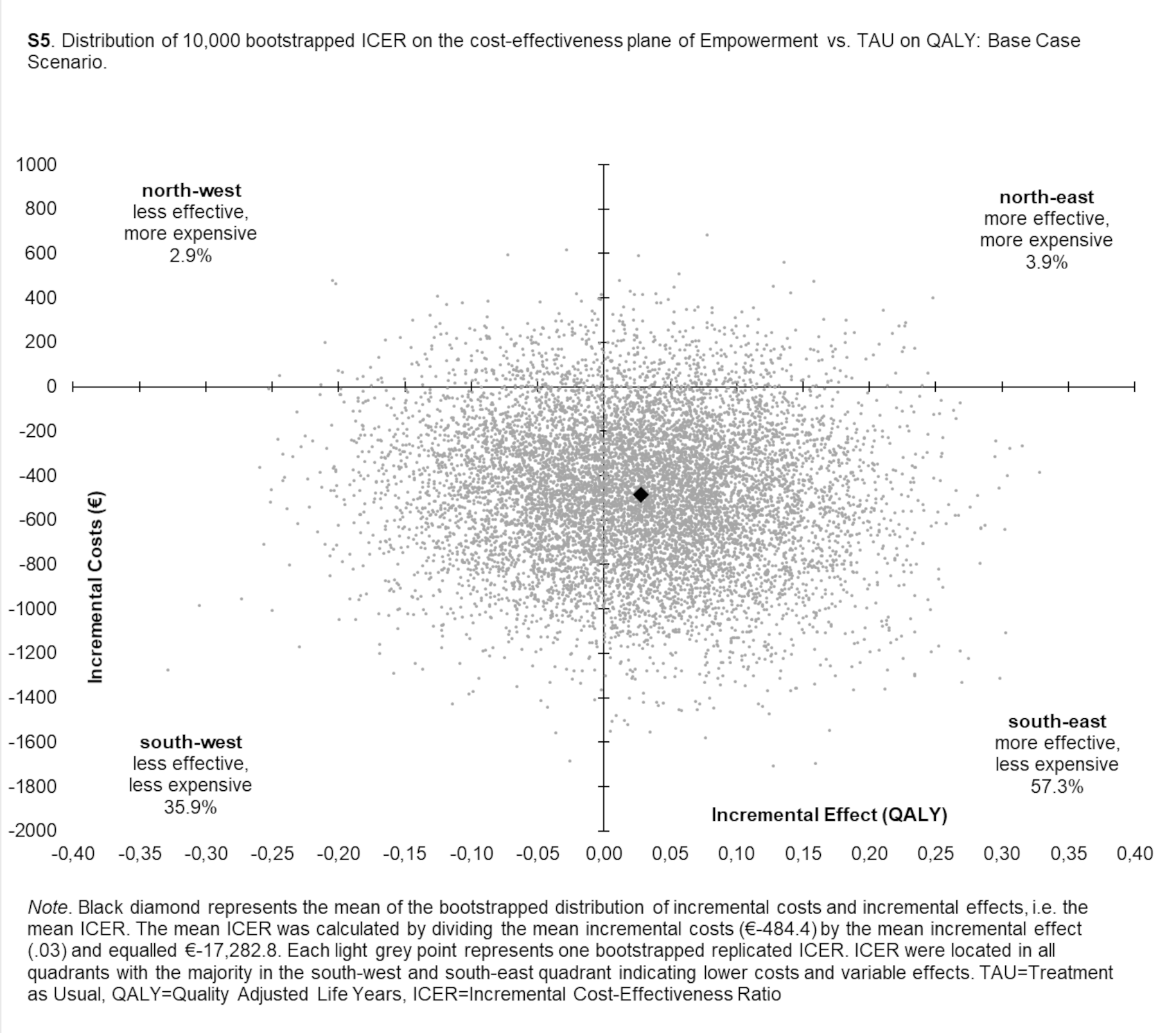

Supplement: Supplementary file 1 [file S2056472423005045sup001.zip › S2056472423005045sup005.tif]
